# Supplementary material for: HIVprotI: an integrated web based platform for prediction and design of HIV proteins inhibitors
Source: J Cheminform. 2018 Mar 9;10:12. doi: 10.1186/s13321-018-0266-y (PMC5845081; doi:10.1186/s13321-018-0266-y)
Supplement: Supplementary file 2 — Additional file 2. Source code of HIVProtI web server. [file 13321_2018_266_MOESM2_ESM.zip › HIVprotI_Source-code/hivprotI/jsme/api_javadoc/index-all.html]

Index


JavaScript is disabled on your browser.


Skip navigation links


- Package
- Class
- Tree
- Deprecated
- Index
- Help

- Prev
- Next

- Frames
- No Frames

- All Classes

A C D E G I J M N O R S U 


## A

addClickHandler(JsClosureJsme) - Method in class export.client.JSME
:   Add a click handler to a JSME instance.

addMouseDownHandler(JsClosureJsme) - Method in class export.client.JSME
:   Add a mouse down handler to a JSME instance.

addMouseOutHandler(JsClosureJsme) - Method in class export.client.JSME
:   Add a mouse out handler to a JSME instance.

addMouseOverHandler(JsClosureJsme) - Method in class export.client.JSME
:   Add a mouse over handler to a JSME instance.

alert(String) - Method in class export.client.JSME
:   Display an alert box with the look and feel of JSME


## C

clear() - Method in class export.client.JSME
:   Same effect as clicking on the clear icon: clear the molecular drawing area


## D

deferredRepaint() - Method in class export.client.JSME
:   Repaint the applet after the browser event loop returns.


## E

export.client - package export.client


## G

getAlldefinedCallBackNames() - Method in class export.client.JSME
:   Return an array containing all possible callback names.

getAllGraphicsString() - Method in class export.client.JSME
:   Returns all graphics representations used in the applet, either a SVG or a VML string
    This is an advanced feature.

getCallBack(String) - Method in class export.client.JSME
:   Generic callback

getCreationIndex() - Method in class export.client.JSME
:   The JSME class keeps tract of the number of instance that have been created.

getHelpURL() - Method in class export.client.JSME


getMenuScale() - Method in class export.client.JSME


getMolecularAreaAntiAlias() - Method in class export.client.JSME
:   Return the antialias setting for the molecular drawing area

getMolecularAreaGraphicsString() - Method in class export.client.JSME
:   Returns the graphics representation used in the molecular area, either a SVG or a VML string
    This is an advanced feature.

getMolecularAreaLineWidth() - Method in class export.client.JSME


getMolecularAreaScale() - Method in class export.client.JSME


getNotifyStructuralChangeJSfunction() - Method in class export.client.JSME
:   Deprecated.

getOffsetHeight() - Method in class export.client.JSME
:   Gets the object's offset height in pixels.

getOffsetSize() - Method in class export.client.JSME
:   Gets the object's offset width and height in pixels.

getOffsetWidth() - Method in class export.client.JSME
:   Gets the object's offset width in pixels.

getParentContainer() - Method in class export.client.JSME
:   Return the parent DOM element that contains myself.

getPasteLabel() - Method in class export.client.JSME
:   See `JSME.setPasteLabel(String)`

getPrePasteJSfunction() - Method in class export.client.JSME


getWebSearchInchiKeyBaseUrl() - Method in class export.client.JSME


## I

initializationError(String) - Method in class export.client.JSME


isDepictMode() - Method in class export.client.JSME


isVisible() - Method in class export.client.JSME
:   Returns whether the given element is visible in a way consistent with
    `JSME.setVisible(boolean)`.


## J

jmeFile() - Method in class export.client.JSME


JSME - Class in export.client


JSME(String, String, String) - Constructor for class export.client.JSME


JSME(String) - Constructor for class export.client.JSME


JSME(String, String, String, JavaScriptObject) - Constructor for class export.client.JSME


JSME(String, JavaScriptObject) - Constructor for class export.client.JSME


## M

molFile(boolean) - Method in class export.client.JSME


## N

nonisomericSmiles() - Method in class export.client.JSME


## O

options(String) - Method in class export.client.JSME
:   options is a comma separated value list - same as original JME


## R

read\_MOL\_SDF\_RXN(String) - Method in class export.client.JSME
:   Read a molecular structure encoded in MOL or SDF format, or a reaction encoded in RXN format.

readGenericMolecularInput(String) - Method in class export.client.JSME


readMolecule(String) - Method in class export.client.JSME
:   Read a molecular structure encoded in JME format

readMolFile(String) - Method in class export.client.JSME
:   Read a molecular structure encoded in MOL format, or a reaction encoded in RXN format

repaint() - Method in class export.client.JSME
:   Redraw the whole applet if it is visible

replaceAllAppletsByJSME() - Static method in class export.client.JSME
:   Scan the DOM for DIV's containing an attribute code="JME.class" and install a running JSME.

replaceAllAppletsByJSME(JsClosure) - Static method in class export.client.JSME
:   Scan the DOM for DIV's containing an attribute code="JME.class" and install a running JSME.

reset() - Method in class export.client.JSME


resetAtomColors(int) - Method in class export.client.JSME
:   Reset background colors

runAfterBrowserEventLoopReturns(JavaScriptObject) - Static method in class export.client.Utils
:   Run some Javascript code after the browser event loop returns.

runAfterFixedDelay(JavaScriptObject, int) - Static method in class export.client.Utils
:   Run some Javascript code after the browser event loop returns.


## S

safeLog(String) - Static method in class export.client.Utils
:   console.log tested on Chrome with success On IE: the console is not
    available unless debugger is on: causes an exception.

setAction(int) - Method in class export.client.JSME
:   select one of the menu action without having to click on the menu.

setAfterDepictEditToggleEventHandler(JsClosureJsme) - Method in class export.client.JSME


setAfterPasteCallback(JavaScriptObject) - Method in class export.client.JSME
:   Deprecated.

setAfterStructureModifiedCallback(JavaScriptObject) - Method in class export.client.JSME
:   Deprecated.

setAntialias(boolean) - Method in class export.client.JSME
:   Set globally antialias

setAtomBackgroundColors(int, String) - Method in class export.client.JSME


setAtomMolecularAreaFontSize(float) - Method in class export.client.JSME


setAtomToHighLight(int, int) - Method in class export.client.JSME


setBeforePasteCallback(JavaScriptObject) - Method in class export.client.JSME
:   Deprecated.

setCallBack(String, JavaScriptObject) - Method in class export.client.JSME
:   Generic call back.

setHeight(String) - Method in class export.client.JSME
:   Sets the object's height.

setHelpURL(String) - Method in class export.client.JSME
:   set the url to display the JSME help page.

setMenuScale(double) - Method in class export.client.JSME


setMolecularAreaAntiAlias(boolean) - Method in class export.client.JSME
:   Set antialias for the molecular drawing area

setMolecularAreaLineWidth(float) - Method in class export.client.JSME
:   Line width in pixel

setMolecularAreaScale(double) - Method in class export.client.JSME


setNotifyAtomHighLightChangeJSfunction(String) - Method in class export.client.JSME
:   Deprecated.

setNotifyStructuralChangeJSfunction(String) - Method in class export.client.JSME
:   Deprecated.

setPasteLabel(String) - Method in class export.client.JSME
:   Set the label of the context popup menu for pasting string, instead of using the default label.

setPrePasteJSfunction(String) - Method in class export.client.JSME
:   Deprecated.

setSize(String, String) - Method in class export.client.JSME
:   Sets the object's size.

setSize(int, int) - Method in class export.client.JSME
:   Sets the object's size.

setSize(String, String, boolean) - Method in class export.client.JSME
:   Sets the object's size.

setSize(int, int, boolean) - Method in class export.client.JSME
:   Sets the object's size.

setSubstituent(String) - Method in class export.client.JSME
:   Select the next FG to be used.

setTemplate(String, String) - Method in class export.client.JSME
:   Provide a template, a molecular fragment with one marked atom, in JME format.

setVisible(boolean) - Method in class export.client.JSME
:   Shows or hides the given element.

setWebSearchInchiKeyBaseUrl(String) - Method in class export.client.JSME
:   Set the base url for inchi key search.

setWidth(String) - Method in class export.client.JSME
:   Sets the object's width.

showInfo(String) - Method in class export.client.JSME
:   Display a message in the bottom bar.

smiles() - Method in class export.client.JSME


## U

Utils - Class in export.client


Utils() - Constructor for class export.client.Utils

A C D E G I J M N O R S U

Skip navigation links


- Package
- Class
- Tree
- Deprecated
- Index
- Help

- Prev
- Next

- Frames
- No Frames

- All Classes
